# Which is the best repair of articular-sided rotator cuff tears: a meta-analysis

**DOI:** 10.1186/s13018-015-0224-6

**Published:** 2015-05-28

**Authors:** Lin Sun, Qiang Zhang, Heng’an Ge, Yeqing Sun, Biao Cheng

**Affiliations:** Department of Orthopedics, Shanghai Tenth People’s Hospital, Tongji University, School of Medicine, 301 Yanchang Middle Road, Shanghai, 200072 China; First Clinical Medical College, Nanjing Medical University, Nanjing, China; Department of Orthopedics, Changzhou No. 2 People’s Hospital, Nanjing Medical University, Jiangsu, China

**Keywords:** Partial-thickness rotator cuff tears, Articular-sided, Trans-tendon technique, Tear conversion and repair technique

## Abstract

**Background:**

Tear conversion followed by repair and trans-tendon techniques have been widely used for partial-thickness rotator cuff tears. Both of them showed favorable results with regard to the management of articular-sided partial-thickness rotator cuff tears (PTRCTs) of more than 50 % thickness. However, controversy continues with the best management. This study aims to compare the clinical outcomes between the two techniques.

**Methods:**

The PubMed, Embase, and Cochrane library databases were searched for relevant studies published before October 1, 2014. Studies that clearly reported a comparison between the two procedures were selected. The American Shoulder and Elbow Surgeons scale (ASES) and the re-tear rate were evaluated. Statistical analysis was performed using the special meta-analysis software called “Comprehensive Meta Analysis”.

**Results:**

Final meta-analysis after the full-text review included four studies about tear conversion followed by repair and seven studies about trans-tendon technique. The trans-tendon technique showed no significant difference with the tear conversion followed by repair technique with regard to the ASES scale (*P* = 0.69). But the re-tear rate (*P* < 0.05) was markedly lower in the trans-tendon technique group than the tear conversion and repair technique group.

**Conclusion:**

In conclusion, the meta-analysis suggests that the trans-tendon technique is better than the tear conversion followed by repair technique with regard to the management of articular-sided PTRCTs of more than 50 % thickness in the re-tear rate aspect.

**Electronic supplementary material:**

The online version of this article (doi:10.1186/s13018-015-0224-6) contains supplementary material, which is available to authorized users.

## Introduction

Partial-thickness rotator cuff tears (PTRCTs) may occur on the articular side, within the tendon, or on the bursal side, with the articular-sided tears being 2–3 times more common than bursal-sided tears [1, 2]. Since partial-thickness tears do not have natural integrity potential and may progress to full-thickness tears, operative intervention is typically indicated for patients with persistent pain and disability symptoms, instead of failed conservative management [1, 3]. Several techniques have been introduced for the repair of PTRCTs, including acromioplasty alone, debridement of the partial-thickness tear with or without acromioplasty, trans-tendinous repair, or conversion of the lesion to a full-thickness tear followed by repair. Nowadays, the trend is to repair lesions involving more than 50 % of the tendon thickness with the conversion and repair technique or trans-tendinous technique. The tear conversion and repair technique is a traditional method, and satisfactory clinical outcomes have been reported [3, 4]. However, others advocate the trans-tendon repair technique as the tendon integrity, native footprint, and biomechanical properties are better restored with the intact bursal-sided rotator cuff tendon [5, 6]. Controversy continues with the best management of PTRCTs and few studies of high level of evidence exist.

The purpose of this study was to conduct a meta-analysis to compare the two techniques for treating articular-sided PTRCTs of more than 50 % thickness.

## Methods

The PubMed, Cochrane library, and Embase databases were searched independently by two investigators (QZ and LS) to retrieve relevant studies published before October 1, 2014. The search criteria “partial thickness rotator cuff tears” were used in text word searches. The “related articles” function was used to broaden the search. The reference lists of the selected articles were also manually examined to find relevant studies that were not discovered during the database searches.

We selected any studies that reported outcomes after the operation of articular-sided PTRCTs of more than 50 % thickness. All titles, abstracts, and full papers of potentially relevant studies were assessed for eligibility. When several reports from the same study were published, only the most recent or informative one was included in this meta-analysis.

Inclusion criteria

1. Language: English
2. Arthroscopic repair
3. Greater than 12-month minimum follow-up
4. Follow-up examination presenting at least one of the following matched outcomes: American Shoulder and Elbow Surgeons scale (ASES) score, re-tear rate

Exclusion criteria

1. Less than 12-month minimum follow-up
2. Studies not reporting outcomes of articular-side PTRCTs of more than 50 % thickness
3. Studies including open or mini-open procedures

### Data extraction

The data extraction of all variables and outcomes of interest were performed independently by 2 readers (QZ and LS). Disagreements were resolved through discussion and consensus. Taking into the existence of both randomized and non-randomized studies, we considered that the methodological quality of the included studies should be assessed by the Quality Index. The Quality Index, which consisted of 27 items distributed between five sub-scales, was appropriate for assessing both randomized and non-randomized studies [7]. Matched outcomes were checked throughout the papers. The ASES scale and re-tear rate were the matched outcome and were extracted from all the studies included. In addition, we extracted data on clinical design, country of study, number of participants, and mean follow-up. If articles reported insufficient data, we contacted corresponding authors for additional information.

### Statistical analysis

The statistical analysis was performed using the meta-analysis software called “Comprehensive Meta Analysis”. Continuous values (the ASES scores) was calculated by comparing their means (*t* test). The re-tear rate was compared by *χ*^2^ test. All of the effect sizes were calculated using a random-effects model. Scores (baseline to follow-up) were compared by calculating the standard difference of the means (SDM). All of the results were presented as forest plots. A 95 % confidence interval was given for each effect size. Heterogeneity is expressed as *I*^2^. This value ranges from 0 % (complete consistency) to 100 % (complete inconsistency).

## Results

### Literature search

**Fig. 1:**
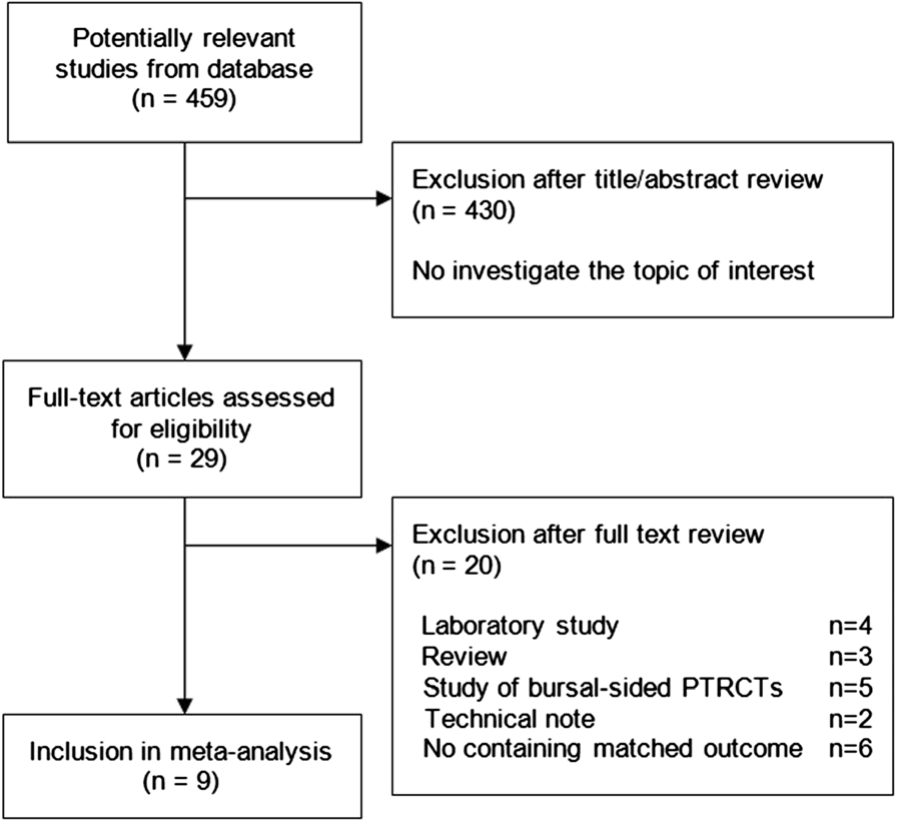
Search strategy flow diagram

The initial literature search retrieved 459 relevant articles (duplicates were discarded). Four hundred thirty articles were excluded for not investigating the topic of interest after carefully screening the titles and abstracts. Then, full publication review was performed, and 20 articles were excluded (4 laboratory studies, 3 reviews, 5 studies of bursal-sided PTRCTs, 2 technical note, 6 studies for not containing matched outcomes, such as the ASES scores and re-tear rate), which left 9 studies for this meta-analysis [4, 7–14]. Final meta-analysis after the full-text review included four studies about tear conversion followed by repair technique and seven studies about trans-tendon technique. Five of the trans-tendon techniques showed the medial row only with one or two suture anchors, and the other two described the medial row combined with pushlock anchors lateral to the greater tuberosity. Flowcharts describing the study selection are in Fig. 1.

A total of 323 patients (99 for tear conversion and 224 for trans-tendon) were enrolled in the studies. The key characteristics of the included studies are summarized in Table 1. All the studies involved patients with articular-sided PTRCTs of more than 50 % thickness. Among the included studies, seven studies investigated the ASES scale and five studies investigated re-tear rate.

**Table 1:** The characteristics of the included studies

Study	Year	Country	Study design	Patients	Intervention	Sample size	Mean follow-up	Mean age	Gender (male/female)	Matching outcome measures
Allen Deutsch	2007	USA	Prospective cohort study	Articular side partial-thickness rotator cuff tears (>50 %)	TCaR	33	38 months	48	22/11	ASES
Castricini R	2009	Italy	Retrospectively cohort study	Articular side partial-thickness rotator cuff tears (>52 %)	TTR	31	33 months	53.3	16/15	Re-tear rate
Jaideep J. Iyengar	2010	USA	Retrospectively cohort study	Articular side partial-thickness rotator cuff tears (>52 %)	TCaR	14	24 months	57.5	Unclear	Re-tear rate
Young-Jin Seo	2011	Korea	Prospective cohort study	Articular side partial-thickness rotator cuff tears (>50 %)	TTR	24	12 months	51	14/10	ASES
Sang-Jin Shin	2012	Korea	RCT	Articular side partial-thickness rotator cuff tears (>50 %)	TTR	24	31 months	53	10/14	ASES, Re-tear rate
Sang-Jin Shin	2012	Korea	RCT	Articular side partial-thickness rotator cuff tears (>50 %)	TCaR	24	31 months	57	13/11	ASES, Re-tear rate
Xavier A. Duralde	2012	USA	Retrospectively cohort study	Articular side partial-thickness rotator cuff tears (>50 %)	TTR	50	38 months	48.7	Unclear	ASES
Kyung Cheon Kim	2013	Korea	Prospective cohort study	Articular side partial-thickness rotator cuff tears (>50 %)	TTR	32	17.4 months	51.8	16/16	ASES
Francesco Franceschi	2013	Italy	RCT	Articular side partial-thickness rotator cuff tears (>50 %)	TCaR	28	39 months	55.6	13/15	ASES, Re-tear rate
Francesco Franceschi	2013	Italy	RCT	Articular side partial-thickness rotator cuff tears (>50 %)	TTR	32	38 months	57.3	18/14	ASES, Re-tear rate
Sung-Jae Kim	2013	Korea	Case–control study	Articular side partial-thickness rotator cuff tears (>50 %)	TTR	29	24 months	59.1	10/19	ASES, Re-tear rate

*TCaR* tear conversion and repair, *TTR* trans-tendon repair

Table 2 summarizes the methodological quality of the included studies. The mean score of the nine studies’ methodological quality was 12.55. Two studies were RCTs with a relatively high score. The prospective cohort study, retrospectively cohort study, and case control study were included, and they got lower scores.

**Table 2:** The methodological quality of the included studies

Item	Deutsch 2007	Kim 2013	Franceschi 2013	Kim 2013	Duralde 2012	Shin 2012	Seo 2011	Iyengar 2010	Castricini 2009
1) Is the hypothesis/aim/objective of the study clearly described?	Y	Y	Y	Y	Y	Y	Y	Y	Y
2) Are the main outcomes to be measured clearly described in the Introduction or Method’s section?	Y	Y	Y	Y	Y	Y	Y	Y	Y
3) Are the characteristics of the patients included in the study clearly described?	Y	Y	Y	Y	N	Y	N	Y	N
4) Are the interventions of interest clearly described?	N	Y	Y	N	N	Y	N	N	N
5) Are the distributions of principal confounders in each group of subjects to be compared clearly described?	N	N	N	N	N	N	N	N	N
6) Are the main findings of the study clearly described?	Y	Y	Y	Y	Y	Y	Y	Y	Y
7) Does the study provide estimates of the random variability in the data for the main outcomes?	N	Y	Y	Y	Y	Y	Y	Y	Y
8) Have all important adverse events that may be a consequence of the intervention been reported?	N	N	N	N	N	N	N	N	N
9) Have the characteristics of patients lost to follow-up been described?	Y	Y	Y	Y	Y	Y	Y	Y	Y
10) Have actual probability values been reported (e.g., 0.035 rather than <0.05) for the main outcomes except where the probability value is less than 0.001?	Y	Y	Y	Y	Y	Y	Y	Y	Y
11) Were the subjects asked to participate in the study representative of the entire population from which they were recruited?	U	U	U	U	U	U	U	U	U
12) Were those subjects who were prepared to participate representative of the entire population from which they were recruited?	U	U	U	U	U	U	U	U	U
13) Were the staff, places, and facilities where the patients were treated representative of the treatment the majority of patients receive?	U	U	U	U	U	U	U	U	U
14) Was an attempt made to blind study subjects to the intervention they have received?	N	N	U	N	N	U	N	N	N
15) Was an attempt made to blind those measuring the main outcomes of the intervention?	N	N	Y	N	N	Y	N	N	N
16) If any of the results of the study were based on “data dredging”, was this made clear?	Y	Y	Y	Y	Y	Y	Y	Y	Y
17) In trials and cohort studies, do the analyses adjust for different lengths of follow-up of patients, or in case–control studies, is the time period between the intervention and outcome the same for cases and controls?	N	Y	N	N	N	N	Y	N	N
18) Were the statistical tests used to assess the main outcomes appropriate?	Y	Y	Y	Y	Y	Y	Y	Y	Y
19) Was compliance with the intervention/s reliable?	Y	Y	Y	Y	Y	Y	Y	Y	Y
20) Were the main outcome measures used accurate (valid and reliable)?	Y	Y	Y	Y	Y	Y	Y	Y	Y
21) Were the patients in different intervention groups (trials and cohort studies) or were the cases and controls (case–control studies) recruited from the same population?	N	Y	Y	N	N	Y	N	N	N
22) Were study subjects in different intervention groups (trials and cohort studies) or were the cases and controls (case–control studies) recruited over the same period of time?	N	Y	Y	N	N	Y	N	N	N
23) Were study subjects randomized to intervention groups?	N	N	Y	N	N	Y	N	N	N
24) Was the randomized intervention assignment concealed from both patients and health care staff until recruitment was complete and irrevocable?	N	N	U	N	N	U	N	N	N
25) Was there adequate adjustment for confounding in the analyses from which the main findings were drawn?	N	N	N	N	N	N	N	N	N
26) Were losses of patients to follow-up taken into account?	N	N	N	N	N	Y	Y	Y	N
27) Did the study have sufficient power to detect a clinically important effect where the probability value for a different being due to chance is less than 5 %?	0	0	0	0	0	0	0	0	0
Score	10	15	16	11	10	17	12	12	10

*Y* yes, *N* No, *U* unable to determine

### Main analysis

**Fig. 2:**
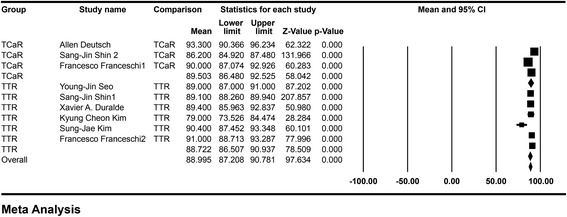
Difference of the ASES scale: the *forest plots* present the mean ASES score of each study. Each *square* represents the individual study’s mean score with a 95 % CI indicated by the *horizontal lines*. Number of included studies: TCaR, *n* = 3; TTR, *n* = 6. Mean TCaR, 89.503; TTR, 88.722. Heterogeneity (*I* ^2^): TCaR = 45.573, TTR = 39.308. Significance: *P* = 0.69

**Fig. 3:**
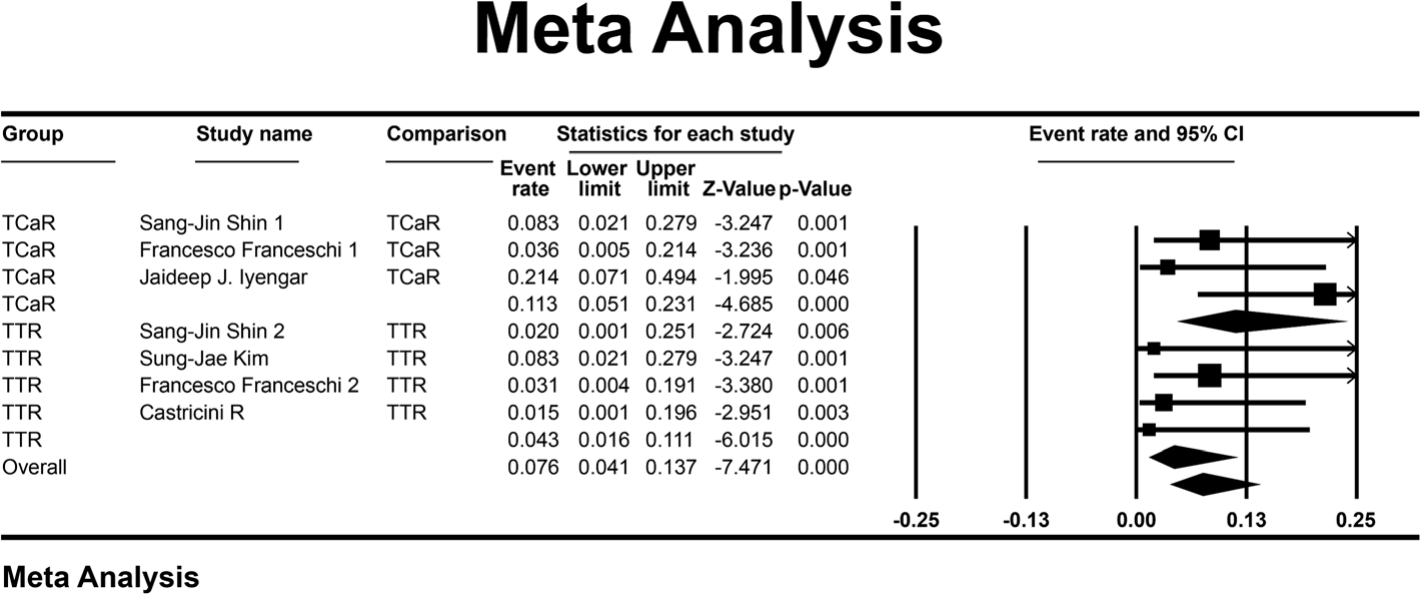
Difference of the re-tear rate: the *forest plots* present the mean re-tear rate of each study. Each *square* represents the individual study’s mean rate with a 95 % CI indicated by the *horizontal lines*. Number of included studies: TCaR, *n* = 3; TTR, *n* = 4. Mean TCaR, 0.105; TTR, 0.043. Heterogeneity (*I* ^2^): TCaR = 34.3, TTR = 0. Significance: *P* <0.001

Figures 2 and 3 summarize the outcomes of the main meta-analysis. With regard to the ASES scale, no statistical difference was found between the Tear Conversion and Repair (TCaR) technique (mean = 89.503, 95 % CI 86.480–92.525) and the Trans-Tendon Repair (TTR) technique (mean = 88.722, 95 % CI 86.507–90.937) (*P*=0.69, Fig. 2). Significant heterogeneity was found between the groups (*P* = 0.025), so the random effect model was used.

With regard to the re-tear rate comparison, TCaR technique (mean = 0.113, 95 % CI 0.051–0.231) was statistical higher than the TTR technique (mean = 0.043, 95 % CI 0.016–0.111) (*P* < 0.05, Fig. 3). No significant heterogeneity was found between the groups (*P* = 0.129), so the fixed effect model was used.

### Publication bias

The funnel plots demonstrated no visual evidence of publication bias.

## Discussion

Partial-thickness tears of the rotator cuff happened commonly, with the potential to cause significant pain and disability. In the older patient population, tears typically occur on the articular side of the supraspinatus tendon, near its insertion onto the greater tuberosity. Biomechanical studies have shown that in the presence of a partial-thickness tear, the strain patterns within the remaining intact rotator cuff are altered, potentially predisposing the tissue to tear propagation [15–18]. Clinical evidence also show the progression of partial-thickness tears [19]. As a result, surgery is usually needed. New techniques such as acromioplasty alone and arthroscopic debridement with or without acromioplasty have made it successful to release pain and improve the function of the patients. As the development of surgical treatments, two repair methods were mostly employed, the trans-tendon technique and the repair after the tear completions. The clinical outcomes of these techniques in respective articles were various, making it difficult to define the most appropriate management for PTRCTs.

In general, the technique used for the repair of these tears involves completion of the tear, followed by rotator cuff repair [20, 21]. Tear completion to full thickness creates an advantageous healing milieu that is akin to an acute full-thickness tear [5]. Although this can lead to good results, completion of the tear potentially excises normal tissue. After this tissue is excised, the normal tissue margin must be brought over and repaired to a lateral bone bed. This can alter the normal footprint of the rotator cuff, remove the degenerative tissue which probably improves tendon-bone healing, and may potentially create a length–tension mismatch of the repaired rotator cuff muscles [6]. Advances in the techniques of arthroscopic repair have led to partially torn rotator cuff repair without tear completion [5, 22–25]. The aim of such trans-tendon techniques is to preserve the lateral bursal-side layer intact and to restore the footprint of the rotator cuff, thus avoiding completion of the tear, followed by cuff repair. Convincing results regarding the improvement of shoulder function and pain relief led to the establishment of these arthroscopic reconstruction techniques. Cadaveric studies also have shown that preservation of the intact rotator cuff tendon provides better biomechanical properties [15, 26]. However, these techniques, pulling a retracted articular layer onto the original footprint may overtighten the bursal aspect and consequently unbalance the tension in the remaining fibers, which may induce faster healing process [9].

The clinical outcomes (such as the ASES score, re-tear rate, and so on) were the most commonly methods to assess the surgical procedure. The above studies expounded the advantages of their own surgical procedure and showed excellent outcomes. Seldom studies with high level of evidence existed regarding the most appropriate surgical procedure of articular-sided partial-thickness rotator cuff tears. With the present meta-analysis, we are able to distinguish that, if there is superiority between the two techniques. In our study, there was no significant difference between the two techniques for the ASES. But there was a significantly lower re-tear rate of the trans-tendon repair technique than tear conversion and repair technique. Therefore, the trans-tendon repair technique showed a trend towards superior outcomes in the re-tear rate aspect with regard to the management of articular-sided partial-thickness rotator cuff tears (PTRCTs) of more than 50 % thickness. However, it was interesting to find that patients treated by trans-tendon repair had more pain and slower shoulder functional improvements during the recovery period despite complete integrity [13]. It needed further researches to confirm this speculation.

Nevertheless, some limitations exist in this meta-analysis. Firstly, only two RCTs (that including both surgical procedures) are included in this study. Other studies’ quality is not as high as the RCTs. Secondly, there are many other modified trans-tendon repair techniques, which is expected to enhance healing condition for repaired tendon. This study did not distinguish among the modified trans-tendon repair techniques which may be able to present better outcomes than tear conversion and repair technique. Lastly, many other clinical outcomes, which can assess the surgical procedures, are not considered in this study.

Although there were many limitations in this meta-analysis, it still can be used to guide future clinical work.

## Conclusion

In conclusion, the meta-analysis suggests that the trans-tendon technique is better than the tear conversion followed by repair technique with regard to the management of articular-sided partial-thickness rotator cuff tears (PTRCTs) of more than 50 % thickness in the re-tear rate aspect.

### Supporting information

Completed Preferred Reporting Items for Systematic Reviews and Meta-Analyses (PRISMA) checklist. Additional file 1: Table S1 presents the completed PRISMA checklist for the meta-analysis.

## Additional file

Additional file 1: Table S1. The completed PRISMA checklist for the meta-analysis.
